# Effect of Clinically Relevant Smear Layers and pH of Universal Adhesives on Dentin Bond Strength and Durability

**DOI:** 10.3290/j.jad.b2838121

**Published:** 2022-03-24

**Authors:** Hüseyin Hatırlı, Kaan Yerliyurt

**Affiliations:** a Assistant Professor, Department of Restorative Dentistry, Faculty of Dentistry, Tokat Gaziosmanpasa University, Tokat, Turkey. Conceptualization, data curation, methodology, formal analysis, wrote, reviewed and edited the manuscript.; b Assistant Professor, Department of Prosthetic Dentistry, Faculty of Dentistry, Tokat Gaziosmanpasa University, Tokat, Turkey. Data curation, methodology, formal analysis, reviewed and edited the manuscript.

**Keywords:** smear layer, universal adhesive, self-etch adhesives, bond strength, microtensile bond strength

## Abstract

**Purpose:**

To evaluate the effects of different smear layers on the microtensile bond strength (µTBS) of a reference two-step self-etch adhesive and two universal adhesives.

**Materials and Methods:**

Mid-coronal dentin of 90 teeth was exposed and divided into three bur groups (coarse diamond, fine diamond, or tungsten carbide). Each bur-prepared group was further divided into three adhesive groups: Clearfil SE Bond (SE, Kuraray Noritake), Single Bond Universal (SB; 3M Oral Care), and G-Premio Bond (GP, GC). After adhesive application, 4-mm-thick resin composites were built up. Half of the teeth in each bur-adhesive group were used in immediate µTBS testing, and the others were tested after thermal aging (n = 5). Rectangular sticks were prepared using a low-speed diamond saw. For each tooth, 6 central sticks were used in the µTBS test. Statistical analysis was performed using three-way ANOVA and Bonferroni tests (α = 0.05).

**Results:**

SE presented higher µTBS than universal adhesives and SB presented higher µTBS than GP regardless of dentin surface preparation and thermal aging (p ˂ 0.05). For SE and SB, the tungsten carbide bur demonstrated higher immediate and aged µTBS than did the extra-fine diamond bur (p ˂ 0.05). The immediate µTBS was similar for GP with all bur types (p ˃ 0.05); the tungsten carbide and extra-fine diamond burs presented higher µTBS than did the coarse-diamond bur after thermal aging (p ˂ 0.05).

**Conclusion:**

Dentin surface preparation and adhesive type had significant effects on µTBS. The smear layer created with an extra-fine diamond or tungsten carbide bur is favorable when mild and ultra-mild self-etch adhesives are used.

Universal adhesives have gained popularity among dentists owing to advantages such as user friendliness, low technique sensitivity, faster application, and applicability in both etch-and-rinse or self-etch modes.^1,10^ This new generation of adhesives has demonstrated favorable bonding performance regardless of the bonding procedure applied.^13,29^ However, in addition to their long-term durability, there are concerns regarding the bond strength of universal adhesives when bonding to different types of smear layers.^20^

Based on the application steps, self-etch adhesives are classified as either 1-step or 2-step self-etch adhesives.^28^ They are further subdivided based on their acidity (strong, semi-strong, mild, and ultra mild).^15^ In addition to the application steps and pH values, differences in the application procedures, formulation, and ingredients influence the immediate and long-term adhesive performance of self-etch adhesives.

During cavity preparation, a smear layer (SL) is produced on the enamel and dentin surfaces using rotary or hand instruments. The composition, thickness, and morphological features of SL differ with respect to the tooth substrate, irrigation method, and instruments used.^25^ In addition, the preparation of dentin surface with different burs or abrasives results in different SLs, qualitatively as well as quantitatively, which affects the bonding efficacy of the self-etch adhesives.^6^ For tooth-surface standardization, specimen surfaces are commonly prepared using silicon carbide (SiC) papers in in-vitro studies, which are considered to be clinically irrelevant,^20,23^ or by using diamond burs.^21^ Yet in dental practice, mechanical dentin caries removal is performed using tungsten carbide burs. However, data on the bond strength and hybrid layer characteristics of universal adhesives – which are applied to dentin prepared using tungsten carbide burs – are not available to date.

SL can be considered an obstacle to the infiltration of self-etch adhesives and should be adequately dealt with.^15^ The importance of the cavity preparation method has been highlighted in previous studies, owing to the fact that thicker SL impairs the effectiveness of mild and ultra-mild self-etch adhesives.^22,28^ Various in vitro studies have observed the effects of the types of SL on the bonding efficacy of self-etch adhesives to enamel and dentin.^4,6,8,11,20,22,23^ However, there is no consensus on the effect of SL type on the adhesive performance of self-etch adhesives. Two-step self-etch and strong or semi-strong 1-step self-etch adhesives do not appear to be substantially influenced by the type of SL.^8,24^ In contrast, for mild or ultra-mild 1-step self-etch adhesives, the effect of SL type depends on the formulation and pH of the adhesive. Previous studies have reported that thicker SLs have a negative effect of on the bond strength.^8,22^ In addition, the available knowledge on universal adhesives with respect to this aspect is limited.

Therefore, this study aimed to evaluate the effects of clinically relevant SLs on the microtensile bond strength (µTBS) of a reference 2-step self-etch adhesive and two different universal adhesives applied in self-etch mode. The null hypotheses tested were: 1) there is no effect of the type of adhesive, 2) no effect of different SLs created with different burs, and 3) there is no negative effect of thermal aging on the µTBS of the tested adhesives.

## Materials and Methods

### Tooth Selection and Dentin Surface Preparation

The study protocol was approved by the Ethics Committee of the Tokat Gaziosmanpasa University, Faculty of Medicine (No. 20-KAEK-209). Ninety-three extracted human third molars that were free of caries, cracks, or fractures were used in this study. All teeth were stored in an aqueous solution of 0.5% chloramine-T at 4°C and were used within 6 months of extraction.

Ninety teeth that were used for the µTBS test were embedded in a self-curing acrylic resin (Imicryl; Konya, Turkey). Occlusal surfaces of the teeth were cut using a low-speed diamond saw (Microcut 125, Metkon; Bursa, Turkey) under continuous cooling with a water and cutting-fluid mixture (Metcool II, Metkon) to expose the mid-coronal dentin. The dentin surfaces were examined for the presence of enamel or exposure of the pulp. The teeth were randomly divided into three groups (n = 30) according to the following dentin surface preparation procedures:

- Group 1: A cylindrical coarse-grit diamond bur (107–181 µm, 852 FG Meisinger, Hager & Meisinger; Neuss, Germany) at high speed with copious water cooling.
- Group 2: First, the dentin surfaces were prepared as in group 1, followed by a cylindrical extra-fine grit diamond bur (10–36 µm, 852 FG Meisinger) at high speed with copious water cooling.
- Group 3: A cylindrical 8-fluted tungsten carbide bur (HM21R Meisinger) at low speed with the handpiece running at 10,000 rpm with copious water cooling.

Five strokes were applied with light pressure using the burs to create uniform dentin surfaces.^21^

### Adhesive Procedure and Aging

Each bur group was further subdivided into three adhesive subgroups: a: Clearfil SE Bond (SE, Kuraray Noritake; Tokyo, Japan), b: Single Bond Universal (SB, 3M Oral Care; St Paul, MN, USA), and c: G-Premio Bond (GP, GC; Tokyo, Japan). After surface preparation, adhesive procedures were performed immediately. The material compositions and application steps suggested by the manufacturers are listed in Table 1. After adhesive application and polymerization, 4-mm-thick resin composites (Filtek Z250; 3M Oral Care) were built up on the bonded dentin surfaces in 2-mm increments. Each layer was light cured using an LED light-curing unit (20 s, 1000 mW/cm^2^, Valo, Ultradent; South Jordan, UT, USA). Dentin preparation, adhesive application, and resin composite build-up procedures were all performed by the same operator to ensure standardization.

**Table 1:** Adhesives, composition, and application procedures

Adhesive (Manufacturer)	Classification	Composition	pH	General application
Clearfil SE Bond (Kuraray Noritake; Tokyo, Japan)	Two-step mild self-etch	Primer: 10-MDP, HEMA, hydrophilic dimethacrylate, di-camphorquinone, N,N-diethanol-p-toluine, water Bond: 10-MDP, bis-GMA, HEMA, hydrophobic dimethacrylate, di-camphorquinone, N,N-diethanol-p-toluine, silanated colloidal silica	2.0 (primer)	Apply primer for 20 s. Dry with mild air flow. Apply bonding. Gently air dry. Light cure for 10 s.
G-Premio Bond (GC; Tokyo, Japan)	Semi-strong self-etch	10-MDP, 4-META, 10-methacryoyloxydecyl dihydrogen thiophosphate, methacrylate adic ester, distilled water, acetone, photo- initiators, silica fine powder	1.5	Apply using a microbrush. Leave undisturbed for 10 s after application. Dry thoroughly for 5 s with oil-free air under maximum air pressure. Light cure for 10 s.
Single Bond Universal (3M Oral Care; St Paul, MN, USA)	Ultra-mild self-etch	10-MDP, HEMA, silane, dimethacrylate resins, VitrebondTM copolymer, filler, ethanol, water, initiators	2.7	Apply the adhesive to the entire surface and rub it in for 20 s. Gently air dry the adhesive for approximately 5 s for the solvent to evaporate. Light cure for 10 s.

10-MDP: 10-methacryloxydecyl dihydrogen phosphate; HEMA: 2-hydroxyethyl methacrylate; bis-GMA: bisphenol A diglycidyl methacrylate; 4-META: 4-methacryloyloxyethyl trimellitate anhydrate.

After the application of resin composite buildups, the specimens were kept in distilled water at 37°C for 24 h. Half of the teeth in each bur-adhesive group (n = 5) underwent immediate µTBS testing. Before testing, the remaining half were thermally aged in a thermocycler (SD Mechatronik Thermocycler, SD Mechatronik; Westerham, Germany) for 25,000 cycles with 20 s of immersion at each temperature (5°C and 55°C) and a 5-s transfer time between baths.

### µTBS Test

Half of the teeth (n = 45) were used for the immediate µTBS test, while the other half were tested after thermal aging. Each tooth was placed in a low-speed diamond saw (Microcut 125 Precision Cutter, Metkon) perpendicular to the bonding surface and sectioned under continuous cooling with water and a cutting-fluid mixture (Metcool II, Metkon) to obtain rectangular adhesive-dentin sticks (cross-sectional area: 1±0.1 mm^2^). For each tooth, six central sticks were randomly selected for use in the µTBS test; thus, a total of 30 rectangular sticks were tested for each bur-adhesive subgroup. The exact dimensions of the adhesive-dentin sticks were measured using calipers. The specimens were fixed onto a modified Geraldeli’s jig in a µTBS testing apparatus using cyanoacrylate glue. Subsequently, the specimens were subjected to tensile force in a universal testing machine (Shimadzu; Kyoto, Japan) at a cross-head speed of 1 mm/min. The µTBS was calculated in MPa by dividing the fracture force by the bonded area. The Academy of Dental Materials guidelines on the µTBS test protocol were strictly followed during specimen preparation and testing.^2^

### Failure Mode Analysis

All the fractured specimen surfaces were examined stereomicroscopically (Stemi C-2000, Zeiss; Oberkochen, Germany) to determine the mode of failure (adhesive interfacial failure, cohesive failure in resin composite, cohesive failure in dentin, or mixed failure). Two representative samples of each experimental group with a µTBS close to the mean were selected and subsequently imaged using field emission scanning electron microscopy (FE-SEM, Mira 3 XMU, Tescan; Brno, Czech Republic).

### FE-SEM Observation of Prepared Dentin Surfaces

Three teeth were used to observe the dentin surfaces prepared with different burs. Mid-coronal dentin slices (2 mm thick) were obtained from the teeth using a low-speed diamond saw (Microcut 125 Precision Cutter, Metkon). Further, transversal grooves 1 mm deep were prepared with a high-speed cylindrical bur (Coarse, 852FG Meisinger) on the back side of the smear-layer surface. Thereafter, the dentin slices were randomly allocated to three bur groups. The SL was made with burs in the same way described for specimen preparation. Afterwards, specimens were prepared for FE-SEM according to the protocol described by Perdigao et al^18^ as follows. The specimens were fixed in 2.5 glutaraldehyde for 24 h and dehydrated in increasing concentrations of ethanol (50%, 60%, 70%, 85%, 95%, and 100%) twice per concentration, 15 min each time. Then the specimens were chemically dried with hexamethyldisilazane for 10 min and allowed to air dry for 10 min. Finally, the dentin slices were divided into halves through grooves with a hammer and blade. The specimens were sputter-coated with Pt-Pd and observed using FE-SEM (Mira 3 XMU, Tescan).

### FE-SEM Observation of Interfacial Structure

The morphology of the adhesive interfaces was observed using five central rectangular sticks randomly selected from each specimen of each group. The rectangular sticks were fixed to epoxy resin molds to expose the upper surfaces. Specimens were prepared for SEM observation according to the protocol described by Ting et al.^26^ The specimens were ground with 600-, 800-, and 1000-grit SiC papers (Buehler; Lake Bluff, IL, USA) and diamond polishing paste (Ultradent). Specimen surfaces were treated with 1 M 5% HCl for 30 s followed by 5% NaOCl for 5 min and rinsed with distilled water. After drying, the specimens were sputter-coated with Pt-Pd and observed using FE-SEM (Mira 3 XMU, Tescan).

### Statistical Analysis

Statistical analyses were performed using SPSS version 19 (IBM SPSS; Armonk, NY, USA). The µTBS data were analyzed using three-way ANOVA to determine the influence of thermal aging, dentin surface preparation, and adhesive type. The Bonferroni test was used for pair-wise post-hoc comparisons between groups (α = 0.05).

## Results

### Microtensile Bond Strength

No pre-test failures occurred in the present study. The mean µTBS (±SD) values are presented in Table 2. Three-way ANOVA revealed that adhesive (F = 438.951, p ˂ .001) and dentin surface preparation (F = 22.173, p ˂ .001) had significant effects on µTBS, whereas the effect of thermal aging (F = 0.773, p = 0.381) was not significant. Only a slightly significant interaction was observed between the adhesive and dentin surface preparation (F = 2.571, p = 0.037).

**Table 2:** Microtensile bond strengths (µTBS) ± SD in MPa to dentin

Bur	Clearfil SE Bond		Single Bond Universal		G-Premio Bond	
	Immediate	Aged	Immediate	Aged	Immediate	Aged
Coarse-grit diamond	52.3 ± 5.1^A,a^	52.1 ± 5.3^A,a^	42.8 ± 5.7^A,b^	41.0 ± 4.7^A,b^	29.55 ± 4.8^c^	28.0 ± 6.0^A,c^
Fine-grit diamond	54.1 ± 5.7^A,a^	54.7 ± 6.2^AB,a^	44.9 ± 8.1^AB,b^	42.7 ± 7.5^A,b^	29.1 ± 5.9^c^	33.9 ± 6.4^B,c^
8-fluted tungsten carbide	59.0 ± 1^B,a^	57.4 ± 11.8^B,a^	48.2 ± 10.9^A,b^	51.12 ± 12.02^B,b^	29.1 ± 4.6^c^	33.73 ± 9.1^B,c^

*The superscript lowercase letters indicate significant differences between burs (in rows); the superscript uppercase letters indicate significant differences between adhesives (in columns) (p ˂ 0.05).

The reference two-step self-etch adhesive, SE, exhibited higher µTBS than did the universal adhesives SB and GP (p ˂ 0.05), of which SB had higher µTBS than did GP, irrespective of dentin surface preparation and thermal aging (p ˂ 0.05). For each adhesive, the immediate µTBSs of the groups were similar to that of post-aging µTBS, except for GP applied on extra-fine bur- and tungsten-carbide-prepared dentin surfaces, which presented higher µTBS values after thermal aging (p ˂ 0.05).

For SE and SB, tungsten carbide bur groups exhibited both higher immediate and aged µTBS values than did the extra-fine grit diamond bur (p ˂ 0.05). The difference between the coarse and extra-fine burs was not significant (p ˃ 0.05). In addition, the immediate µTBSs were similar for GP irrespective of the burs used (p ˃ 0.05). However, for GP, the 8-fluted tungsten carbide bur and extra-fine diamond bur groups presented higher µTBSs than did the coarse-grit diamond bur group after thermal aging (p ˂ 0.05).

### Fracture Mode Analysis

**Fig 1:**
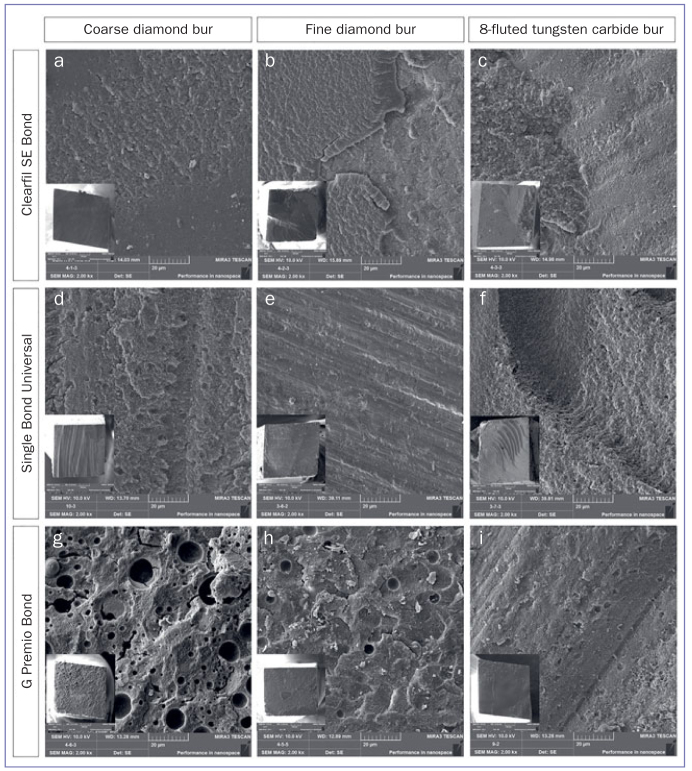
Representative FE-SEM images of failure mode in groups at 2000X (inserts: 150X) original magnification. SE bonded to coarse diamond-prepared dentin (a); SE bonded to fine diamond-prepared dentin (b); SE bonded to coarse 8-fluted tungsten carbide-prepared dentin (c); SU bonded to coarse diamond-prepared dentin (d); SU bonded to fine diamond-prepared dentin (e); SU bonded to coarse 8-fluted tungsten carbide-prepared dentin (f); GP bonded to coarse diamond-prepared dentin (g); GP bonded to fine diamond-prepared dentin (h); GP bonded to coarse 8-fluted tungsten carbide prepared dentin (i).

The number of fracture modes for each group is listed in Table 3. The most frequent fracture modes were adhesive (333/540) and mixed (131/540). A clear tendency for cohesive failures was observed in the groups that presented higher µTBSs. Cohesive failures mostly occurred in the SE (40/120) and SB (28/120) groups. The main failure mode was adhesive for GP (93/120). When adhesive failures were examined by FE-SEM at 2500X magnification, numerous pores on the entire surface were observed for GP (Fig 1).

**Table 3:** Distribution of fracture modes (A/CC/CD/M)*

Bur	Clearfil SE Bond		Single Bond Universal		G-Premio Bond	
	Immediate	Aged	Immediate	Aged	Immediate	Aged
Coarse-grit diamond	15/3/2/10	14/0/5/11	20/4/1/5	21/0/1/8	25/2/0/3	26/0/0/4
Fine-grit diamond	14/5/4/7	13/4/2/11	21/0/2/7	18/2/6/4	22/1/1/6	25/0/2/3
8-fluted tungsten carbide	11/3/3/13	9/2/7/12	16/5/1/8	17/2/4/7	22/1/1/6	24/0/0/6

*A: adhesive failure; CC: cohesive failure in resin composite; CD: cohesive failure in dentin; M: mixed failure.

### FE-SEM of Prepared Dentin Surface and Interfacial Structure

**Fig 2:**
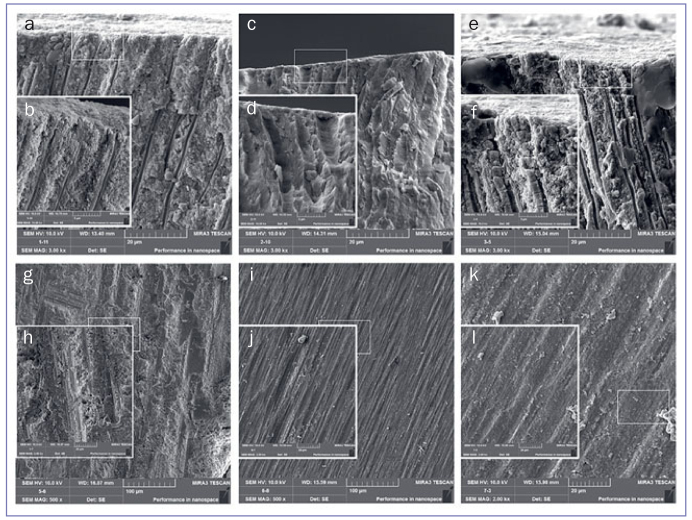
Representative FE-SEM images of dentin smear layers. A longitudinal section of the dentin surface prepared by a coarse diamond bur at original magnifications of (a) 3000X and (b) 10,000X, fine diamond bur at magnifications of (c) 3000X and (d) 10,000X, and 8-fluted tungsten carbide bur at magnifications of (e) 3000X and (f) 10,000X. Dentin surface prepared by a coarse diamond bur at original magnifications of (g) 500X and (h) 2000X, fine diamond bur at magnifications of (i) 500X and (j) 2000X, and an 8-fluted tungsten-carbide bur at original magnifications of (k) 500X and (l) 2000X.

**Fig 3:**
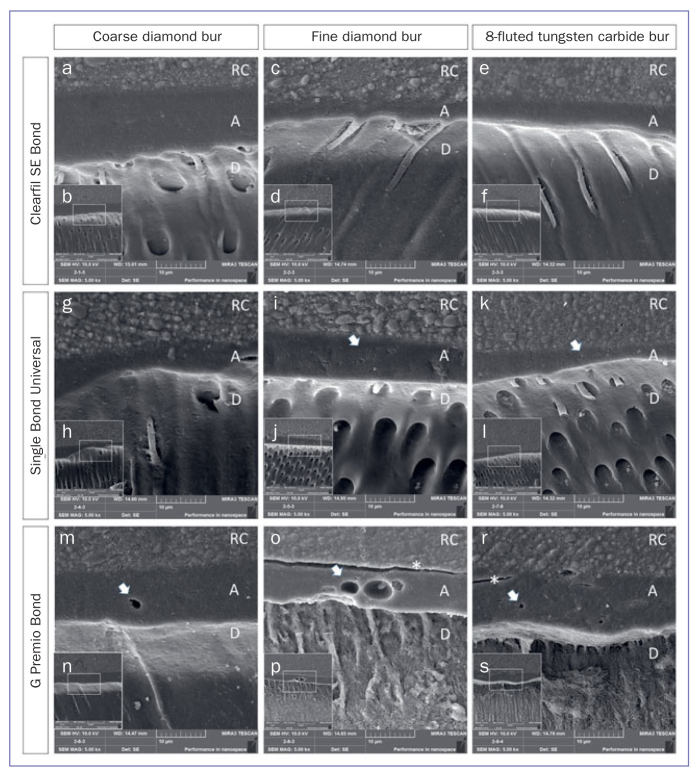
Representative FE-SEM images of adhesive-dentin interfaces of the tested adhesives, according to the bur type dentin surface preparation at an original magnification of 5000X (inserts 2500X). SE bonded to coarse diamond-prepared dentin (a); SE bonded to fine diamond-prepared dentin (c); SE bonded to coarse 8-fluted tungsten carbide-prepared dentin (e); SU bonded to coarse diamond-prepared dentin (g); SU bonded to fine diamond-prepared dentin (i); SU bonded to coarse 8-fluted tungsten carbide-prepared dentin (k); GP bonded to coarse diamond-prepared dentin (m); GP bonded to fine diamond-prepared dentin (o); GP bonded to coarse 8-fluted tungsten carbide-prepared dentin (r). RC: resin composite; A: adhesive layer; D: dentin; white arrows indicate voids; * cracks between adhesive and resin composite.

Representative FE-SEM images of bur-prepared dentin surfaces are presented in Fig 2. The dentin surface prepared with the coarse diamond displayed a rough surface and irregular grooves. The grooves were shorter and narrower when the extra-fine diamond bur was used. The dentin surface prepared with the tungsten-carbide bur exhibited wide, uniform grooves. FE-SEM images revealed that SL covered the dentin surfaces and smear plugs occluded the orifices of dentin tubules. Representative FE-SEM images of adhesive-dentin interfacial structures of each group are shown in Fig 3. For SE, long resin tags inside the dentin tubules were observed using FE-SEM. However, for SB and GP, the resin tags were few and short. In addition, GP presented 1- to 5-µm round gaps, whereas SB presented smaller gaps of up to 1 µm. For GP, in some of the resin composite-adhesive interfaces, line-shaped separations were observed (Figs 1 and 3).

## Discussion

This study assessed the effects of clinically relevant SLs, created using different burs, on the immediate and thermally aged µTBS of a reference 2-step self-etch, a semi-strong, and an ultra-mild universal adhesive. The results of this study revealed that dentin surface preparation and adhesive type had significant effects on µTBS. Therefore, the null hypothesis that there is no effect of 1) type of adhesive and 2) SLs created with different burs was rejected.

The SL can be considered a physical barrier that considerably influences bonding performance,^4,8^ owing to the division of the hybrid layer into an upper, resin-infiltrated demineralized smear layer, and a lower, true hybrid layer.^12^ In addition, SL characteristics are directly affected by the type of rotary instrument, cutting speed, and grit size of the burs used (Fig 2).^12^ To create a clinically relevant SL for bond strength tests, 8-fluted carbide and medium-grit diamond burs for direct bonding and fine-grit diamond burs for indirect bonding are recommended.^2^ When using diamond burs at high speeds, a SL with higher density is created than with SiC papers,^16,22^ and the SL thickness increases with the increasing coarseness of the diamond bur.^21^ Dias et al^6^ observed that diamond burs at high speed produce a rougher surface and that the use of carbide burs can enhance bond strength. However, the effects of diamond and carbide burs on the bond strength and durability of universal adhesives have not been elucidated.

One of the major concerns with self-etch adhesives is the potential interference of the SL created by the bur during cavity preparation. The results of present study revealed that the reference mild 2-step self-etch adhesive (SE) and the ultra-mild universal adhesive (SB) yielded significantly higher µTBS on SL created with a tungsten carbide bur than on SL created with a coarse-grit diamond bur (p ˂ .05). Saikaew et al^20^ stated that the acidic monomers of self-etch adhesives are buffered and their acidity is decreased by the SL. They also confirmed that in the thicker and more compact SL created with regular-grit diamond bur, acidic monomers might not penetrate uniformly. However, the immediate µTBS of the semi-strong universal adhesive (GP) was not affected by the type of SL. This might be explained by the strong acidity of the adhesive.

The pH values of self-etch adhesives can significantly influence the dissolution of the SL and etching of the dentin surface.^24^ Universal adhesives differ from each other in acidity; the bond strength to dentin along with the bond stability have been shown to depend on acidity.^5^ Therefore, this study tested universal adhesives with quite different pH values: a semi-strong (GP; pH: 1.5) and an ultra-mild (SB; pH: 2.7) adhesive, as well as the reference mild two-step self-etch adhesive (SE), according to the classification by Van Meerbeek et al.^15^ The higher pH of the ultra-mild universal adhesive (SB) would be expected to result in reduced etching ability, be less able to etch different SL types, and reduce demineralization of the dentin surface, which is beneficial for micromechanical interlocking. However, consistent with the findings of previous studies, SB showed a higher µTBS than the other universal adhesive, GP, for all SL types.^24^ This might be explained by the differences in ingredients, such as solvent and functional monomer, in addition to the application method. SB contains 2-hydroxyethyl methacrylate (HEMA) and is an ethanol-water based adhesive, whereas GP does not contain HEMA and is an acetone-water based adhesive. HEMA is a hydrophilic monomer contained in many universal adhesives due to it being a good diffusing agent and acting as a co-solvent.^15^ Despite being highly volatile, acetone does not adequately promote water evaporation, because it does not form an azeotrope with water.^4,30^ Numerous round voids and line-shaped separations at composite-adhesive interfaces were observed in the FE-SEM images (Figs 1 and 2) of the GP groups, which can be attributed to the phase separation that occurred due to the absence of HEMA.^27^ These findings might be the reason for existence of mainly adhesive failures along with the lower bond strength of GP groups.^4^ In addition, according to the manufacturer’s instructions, GP was applied for 10 s with an inactive application technique, whereas SB was rubbed in for 20 s. Yoshihara et al^34^ stated that rubbing action promotes dissolution of SL and effective solvent evaporation. Moreover, all the adhesives tested in the present study contain different concentrations of 10-methacryloyloxydecyl dihydrogen phosphate (10-MDP) as a functional monomer. 10-MDP has the ability to chemically interact with hydroxyapatite, and a longer application time with active application through rubbing might foster intimate contact of functional monomers with hydroxyapatite crystals.^4,34^ In other words, for GP, a shorter application time might be insufficient for surface decalcification and adhesive penetration into dentin.^20^ In agreement with the results of previous studies, SE presented higher µTBS than both of the universal adhesives, regardless of the aging procedure and smear layers created with different burs.^9,24^ The separate application of primer and bond in two-step self-etch adhesives can create a more hydrophobic adhesive layer with enhanced mechanical properties.^14,30^ This may explain the significantly higher µTBSs of the SE groups.

Long-term water storage or thermocycling are common aging methods applied prior to bond strength testing of adhesives. However, a comparison of bond strength data obtained after aging vs immediate bond strength as a reference is required. Therefore, immediate and thermally aged µTBS data were assessed in this study. Although the stresses that occurred during thermocycling were expected to decrease the bond strength due to the formation of gaps and crack propagation through the adhesive interface,^7,17^ consistent with the findings of previous studies, thermal aging did not significantly decrease the bond strength of the adhesives.^3,19^ Therefore, the third null hypothesis that there is no negative effect of thermal aging on the bond strength of the tested adhesives cannot be rejected. All the adhesives tested in the present study contain different concentrations of 10-MDP monomer. 10-MDP can facilitate chemical bonding with hydroxyapatite and the formation of nanolayering at the bonding interface.^32,33^ The creation of a durable and mechanically stable adhesive interface with 10-MDP monomer may explain the post-thermocycling results obtained in this study.

## Conclusion

The type of burs used for dentin surface preparation and adhesive had significant effects on the µTBS of 2-step self-etch and universal adhesives applied in self-etch mode. Thermal aging did not negatively affect the bond strength of the adhesives. The SL created with the tungsten-carbide bur vs the coarse diamond bur is advantageous when mild and ultra-mild self-etch adhesives are used. Finishing preparation with the tungsten carbide bur instead of the coarse diamond bur for direct bonding and the extra-fine diamond bur for indirect bonding can be recommended.

## Clinical relevance:

Smear layer type, pH, and formulation of the adhesive can influence the dentin bond strength of self-etch adhesives. Finishing preparation with tungsten carbide burs instead of coarse diamond burs for direct bonding, and extra-fine diamond burs for indirect bonding, can be recommended.
